# Impediments to Progress Toward Polio Eradication During 2014–2024: Effectively Addressing the Current Challenges

**DOI:** 10.3390/vaccines13101060

**Published:** 2025-10-17

**Authors:** Steven G. F. Wassilak, Abdinoor Mohamed, John Paul Bigouette

**Affiliations:** 1 Independent Researcher, Atlanta, GA 30307, USA; 2 Global Immunization Division, Centers for Disease Control and Prevention, Atlanta, GA 30329, USA; wyr5@cdc.gov (A.M.); qdz1@cdc.gov (J.P.B.)

**Keywords:** Global Polio Eradication Initiative, poliomyelitis, immunization, outbreak response, wild poliovirus, vaccine-derived poliovirus, vaccine resistance, vaccine hesitancy, security challenges

## Abstract

When the Global Polio Eradication Initiative (GPEI) began in 1988, the year 2000 target was clearly ambitious. Nonetheless, among 20 countries with endemic wild poliovirus transmission in 2000, only Afghanistan, Nigeria and Pakistan remained endemic in 2014; successful global eradication was anticipated within years. Transmission was interrupted in Nigeria after implementing innovative activities; unfortunately, transmission in Afghanistan and Pakistan has continued into 2025. An additional challenge has been controlling outbreaks and interrupting persistent transmission of circulating vaccine-derived poliovirus type 2 detected after global withdrawal of the use of Sabin strain type 2 oral poliovirus vaccine. The impediments to progress since 2014 are detailed and the challenges that the GPEI must successfully mitigate are reviewed herein. Primary challenges since the acute phase of the COVID-19 pandemic include the loss of a sense of urgency and political will/national ownership in stopping poliovirus transmission, lower childhood routine immunization coverage and the decreased quality of outbreak response campaigns. These facets need to be strengthened. Ongoing security challenges require continued vigilance in affected areas of wild poliovirus-endemic countries as well as in countries with persistent transmission of vaccine-derived poliovirus. Eradication can be achieved if the multiple challenges mentioned are urgently and more effectively mitigated. Decreased funding for the GPEI in 2025 represents the primary, acute challenge that, if not urgently addressed, may allow for the reversal of all progress to date.

## 1. Introduction

The World Health Organization (WHO) Member States’ delegates at the 41st World Health Assembly in 1988 resolved to eradicate poliomyelitis (polio) by 2000 and initiated the Global Polio Eradication Initiative (GPEI) [1]. With substantial progress toward eradication by 2000 and steady progress after that, there were signs by 2014 that the eradication goal was “on the threshold” [2,3,4]. Since 2013, indigenous wild poliovirus (WPV) type 1 (WPV1) has been the sole serotype out of all three circulating [5,6,7]. Indigenous WPV type 2 was certified as eradicated in 2015 (last detection in 1999 in India) and indigenous WPV type 3 in 2019 (last detection in 2012 in Nigeria). With India’s last WPV1 case reported in 2011, the WHO South-East Asian Region was the fourth of six WHO regions to have certified the cessation of transmission of indigenous WPV in 2014 [8]. From 20 countries with endemic WPV in 2000, to 10 in 2001 and to 6 in 2003, only Afghanistan, Nigeria and Pakistan remained endemic in 2014 [3,9]. Certification of the WHO African Region was anticipated by 2017, with no detection of WPV1 transmission in Nigeria in 2015; however, surveillance was virtually non-existent in geographic areas under the control of insurgents [10]. WPV1 cases were again detected in Nigeria in August 2016, requiring vigorous efforts to vaccinate children in insurgent-held areas and surveil poliovirus. With sufficient surveillance data, the WHO African Region was certified WPV-free in 2020 [11].

As endemic WPV1 transmission persists in Afghanistan and Pakistan, the Eastern Mediterranean is the remaining WHO region not WPV-free. Afghan authorities and insurgents have implemented multiple bans on house-to-house vaccination efforts, or any campaigns, since 1996 [12,13,14]. The program in Pakistan has had repeated challenges, particularly following changes in government, and variable program effectiveness [15,16,17]. Both countries experienced a resurgence in cases and widening of affected geographic areas in 2024 [12,18].

Circulating vaccine-derived poliovirus (cVDPV) outbreaks can emerge following prolonged transmission of oral poliovirus vaccine (OPV)-related strains in low-immunity populations. The GPEI coordinated global selective withdrawal of Sabin strain OPV type 2 (OPV2) from use in 2016, i.e., “switching” from trivalent OPV (tOPV, containing Sabin strain types 1, 2, and 3) to bivalent OPV (bOPV, containing types 1 and 3) [19,20,21,22,23,24,25,26]. An additional challenge faced by the GPEI has been interrupting the multiple outbreaks and persistent transmission (>12 months) of cVDPV type 2 (cVDPV2) after the “switch” in April 2016. Monovalent Sabin strain type 2 OPV (mOPV2) was used in response campaigns to control cVDPV2 outbreaks from May 2016 [27]. However, some low-quality mOPV2 outbreak response campaigns (i.e., those ineffective in promptly stopping poliovirus transmission) seeded new cVDPV2 emergences. To mitigate seeding new cVDPV2 outbreaks, a novel OPV2 (nOPV2) was developed to be more genetically stable than Sabin strain OPV2 [28,29,30,31]. The WHO granted Emergency Use Listing of nOPV2 in November 2020 with defined criteria for its initial use [31,32]; nOPV2 outbreak response campaigns began in March 2021 [33,34].

The acute phase of the SARS-CoV-2/COVID-19 pandemic in 2020–2021 upended childhood vaccination services, GPEI OPV campaigns and poliovirus surveillance [35,36,37,38,39]. Unfortunately, the pandemic has had a prolonged effect on immunization activities, contributing to an apparent loss of momentum toward WPV1 eradication and in prioritizing prompt, effective cVDPV2 outbreak responses.

There are several recent summaries of GPEI status and challenges [40,41,42,43]. The GPEI currently faces many long-standing and new impediments in reaching its objectives to eradicate all poliovirus transmission. Our review focuses on the details of impediments to the pace of progress toward eradication since 2014 and suggests the potential means of addressing them to attain a polio-free future. The combination of challenges may be difficult to quickly and effectively address. Our review supplements the 2023 mid-term review by the Independent Monitoring Board (IMB) (of progress under the *Polio Eradication Strategy 2022–2026: Delivering on a Promise* [44]) and the IMB 2024 follow-up report [45,46].

## 2. Materials and Methods

Data on poliovirus surveillance and investigations, outbreaks and supplementary immunization activities (SIAs) in endemic, outbreak and other countries are reported by Member States to the WHO. These data are stored in the WHO Polio Information System, of which all GPEI partners and their analytic collaborators have access; we sourced these data as of 27 March 2025.

Confirmed polio cases are laboratory-positive paralytic cases identified by surveillance for acute flaccid paralysis (AFP) and investigation. However, the <1% proportion of poliovirus (PV) infections that result in paralysis limits the value of tracking case counts over time as the best measure of progress, particularly if there are limitations in AFP surveillance performance [47,48,49]. Systematic sampling of convergent sewage with laboratory testing of those samples for PV (environmental surveillance, ES) supplements AFP surveillance and has greatly enhanced the overall sensitivity of poliovirus surveillance [50,51,52,53,54,55]. When implemented, ES can detect PV transmission within the much larger proportion of infections that are asymptomatic; progress can be monitored by the geographic extent of detection and the proportion of ES samples that are PV-positive. We monitored the number of districts (second subnational administrative entity) in Afghanistan and Pakistan with WPV1 transmission confirmed by WPV1 AFP cases and/or ES-positive samples since 2014. Transmission of cVDPV is considered “persistent” when it continues >12 months from the onset of the first identified case/first ES isolate collection date and “established” when lasting >24 months. A series of fixed-timeframe maps for 2014–2024 indicate countries and areas with cVDPV outbreaks or persistent transmission detected within the prior six months, rolling monthly, by serotype or cocirculation.

Virologic testing results were obtained from the Global Poliovirus Laboratory Network (GPLN) database for January 2014–December 2024 (as of 27 March 2025). Accredited GPLN laboratories perform genomic sequencing for poliovirus isolates of the ~900 nucleotide region coding the VP1 surface protein. Analyses of the sequences provide a means of (1) linking poliovirus transmission lineages in different areas over time, indicating the outcome of population movements, including internationally; (2) grouping related WPV linages into “clusters”, groups of polioviruses sharing ≥95% VP1 sequence identity, to identify the transmission of a given lineage and track progress, where the number of identified active clusters indicates the intensity of transmission; and (3) assessing surveillance quality, as empirically low when ≤98.5% VP1 sequence identity is observed between sequences of detected viruses in a given lineage (“orphan” viruses). Independent cVDPV emergence groups are identified by unique nucleotide substitutions. Outbreaks of cVDPVs are defined by evidence of circulation by county and emergence group [56]. Evidence of circulation is defined as (1) at least one confirmed paralytic case genetically linked with other cases, ES isolations or asymptomatic infection of tested healthy children in the community, or (2) multiple independent ES isolations. Although surveillance limitations prevent precisely identifying where emergence occurred, we consider the country where an emergence group is first detected to be the most likely origin. The timepoint of cVDPV emergence from the parental Sabin OPV strain for each serotype can be inferred from the ~1.1%/year nucleotide substitution rate in the VP1 coding region of polioviruses in replication [57]. The range and median size of cVDPV type 1 and 3 (cVDPV1, cVDPV3) outbreaks and the interquartile ranges (IQRs) of cVDPV1 outbreaks have been calculated.

## 3. Progress Toward Wild Poliovirus Eradication, 2014–2024

### 3.1. Global WPV1 Outbreaks

WPV1 transmission in endemic countries has repeatedly led to international spread. During 2003–2012, there were “waves” of WPV1 transmission originating in Nigeria generally cascading in spread across borders of successive neighboring countries, but sometimes non-contiguously [9,58,59,60,61,62]. International spread during 2013–2014 was marked by five long-distance international importations (without evidence of transmission in countries geographically between evident source country and outbreak country): (1) from Nigeria into Somalia (spreading subsequently into Ethiopia and Kenya); (2) from Cameroon into Equatorial Guinea of WPV originating from Nigeria transmission; (3) from Pakistan into Syria (spreading into Iraq); (4) from Pakistan into Egypt, Israel and Occupied Palestinian Territory (West Bank and Gaza) with silent transmission detected by comprehensive ES sampling; and (5) from Equatorial Guinea into Brazil, with a single isolation from a randomly taken sewage sample. On 5 May 2014, the WHO Director-General (DG) declared the international spread of WPV a Public Health Emergency of International Concern (PHEIC) under the International Health Regulations (2005) (https://www.who.int/news/item/05-05-2014-who-statement-on-the-meeting-of-the-international-health-regulations-emergency-committee-concerning-the-international-spread-of-wild-poliovirus (accessed on 4 September 2025)). The Emergency Committee recommended that all residents and long-term visitors departing from 11 countries and areas (Afghanistan, Cameroon, Ethiopia, Equatorial Guinea, Iraq, Israel and Occupied Palestinian Territories (West Bank and Gaza), Nigeria, Pakistan, Somalia and the Syrian Arab Republic) receive full OPV vaccination (or at least one dose) prior to international travel, if they had received no doses in the prior 12 months. The Emergency Committee continues to convene quarterly; after each meeting the DG has endorsed and issued updated recommendations for countries based on detection of transmission within the prior year. The Committee has extended the review and recommendations to the risks of international spread of cVDPV since the May 2015 report. The DG released the 42nd Emergency Committee recommendations in July 2025 (https://www.who.int/news/item/28-07-2025-statement-of-the-forty-second-meeting-of-the-polio-ihr-emergency-committee. All statements are available at https://www.who.int/groups/poliovirus-ihr-emergency-committee (accessed on 4 September 2025)).

Ignoring cross-border lineage transmission between Afghanistan and Pakistan (in either direction), the most recent international spread from an endemic country has been WPV1 genetically linked to Pakistan, identified in ES samples taken in Iran in 2019 [63] and causing outbreaks in Malawi and Mozambique in 2021–2022 [64,65].

### 3.2. WPV1 Transmission in Endemic Countries

#### 3.2.1. Nigeria

Vaccination coverage of infants with three doses of tOPV provided through routine immunization services (RI; also called essential immunization) had been low nationally in Nigeria since national surveys began, and in northern Nigerian states particularly. Polio eradication OPV SIAs in the northern states were of very low quality up to 2010, evidenced by the extent of continued WPV1 and WPV type 3 (WPV3) transmission [66]. With progress accelerating from 2012 [67,68], WPV1 transmission in 2014 was identified in only two northern states, Kano and Yobe [10]. Following ~48 months without WPV1 detection nationally, four confirmed cases were reported in the northeastern state of Borno, with paralysis onset for two each in July and August 2016. For context, Boko Haram insurgents began seizing control of northeastern Nigerian state territory in 2012. The geographic extent of insurgent-held areas peaked in 2016 in Borno and in parts of Yobe and Adamawa states and other countries bordering the Lake Chad basin [69,70]. With this, health services and disease surveillance activities were virtually stopped. Seizure of control in large areas of Borno sparked displacement of ~2 million persons into camps for internally displaced persons (IDPs) and households in secure areas. The two cases in August 2016 were children recently displaced into IDP camps [69].

With the rediscovery of endemic WPV1 transmission, implementation of innovative measures in Borno began to reach the estimated 469,000 children aged <5 years who remained in insurgent-held areas. These measures included military support for liberation of settlements or the vaccination of children in some insurgent-held areas. Satellite imagery indicated compounds with likely habitation and estimated numbers of residing children; Global Position System (GPS)-enabled mobile phones and Geographic Information Systems tracked vaccination teams’ movements [71,72,73,74,75,76,77,78]. By May 2018, approximately 104,330 children aged <5 years resided in the remaining insurgent-held areas, 42% of whom had received ≥1 dose of bOPV [75]. Most unvaccinated children were located in settlement clusters in two widely separated districts [79,80]. Enhanced AFP surveillance utilized community informants within security-compromised areas who were equipped with mobile telephones [73,77,81]. Children with suspected AFP were allowed by insurgents to temporarily relocate to secure areas for investigation and collection of stool specimens [75,81]. Such intensified case-finding and investigation efforts accumulated sufficient evidence for the WHO African Region to be certified WPV-free.

#### 3.2.2. Afghanistan and Pakistan

**Figure 1:**
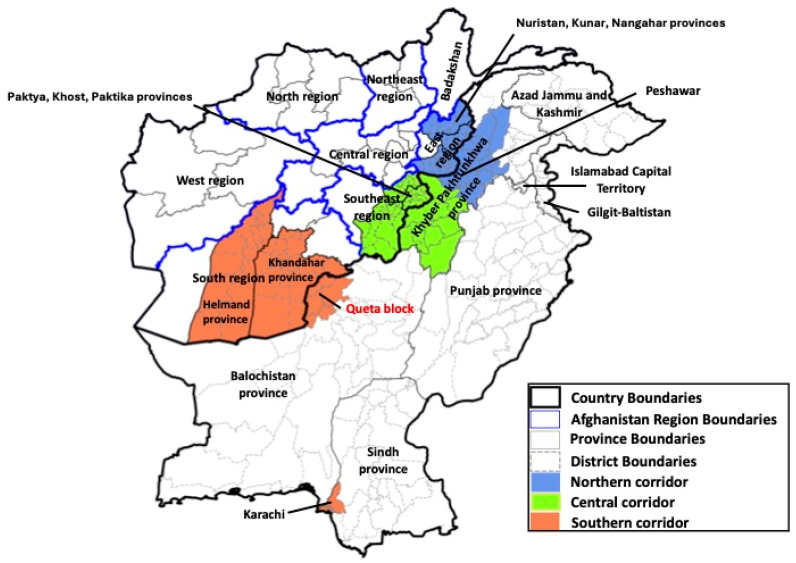
Regions/provinces of Afghanistan and Pakistan, critical districts and cities affected by poliovirus transmission, and intercountry transmission corridors—2014–2024.

These two remaining WPV1-endemic countries constitute a single epidemiologic block, with each having unique factors affecting immunization activities and transmission [82,83]. This includes natural disasters, such as earthquakes in Afghanistan or flooding in Pakistan. Circulation in this block has been related to cross-border travels of highly migrant subpopulations transiting along established bidirectional population movement “corridors”: northern, central and southern (Figure 1) [84,85]. The northern corridor extends from the Afghanistan provinces of Nuristan, Nangarhar and Kunar in the east region to districts in northern Khyber Pakhtunkhwa (KP) province in Pakistan. The central corridor extends from Paktya, Khost and Paktika provinces of the southeast region in Afghanistan to southern KP districts in Pakistan. The southern corridor extends from Helmand and Kandahar provinces in the Afghanistan south region to Balochistan and Sindh provinces, primarily affecting districts in the Quetta block bordering Afghanistan and in Karachi, respectively.

During January 2014–December 2024, the foci of WPV1 transmission along these corridors within Afghanistan were subject to voluntary back-and-forth migration and more recently the forced inward migration from Pakistan, the dynamic political changes toward Taliban control in the affected regions, and their effects on immunization activities [12,13,14,86,87,88,89,90,91,92,93,94].

Multiple operational and socio-cultural issues affect the success of the implementation of SIAs in these countries. Among the socio-cultural issues, there is major suspicion among communities against programs supported by “outsiders” [95]. This was exacerbated by false public sentiments that circulated linking the assassination of Osama bin Laden in 2011 in Pakistan with OPV campaign planning and suspecting OPV vaccinators as being agents of foreign countries (the planning was linked with a bogus hepatitis B vaccination campaign in Abbottabad) [96]. In western parts of Pakistan, this led to targeted intimidation, injury and killing of vaccination workers and their police or military escorts [96,97]. Some of these sentiments persist to date. Misconceptions related to the interpretation of Islamic tenets or the safety of OPV easily spread within communities [96]. In Pakistan, recent widespread use of social media platforms has increased the ease of disseminating misinformation, exacerbating resistance to OPV and other vaccinations among some communities in high-risk areas [98,99].

**Figure 2:**
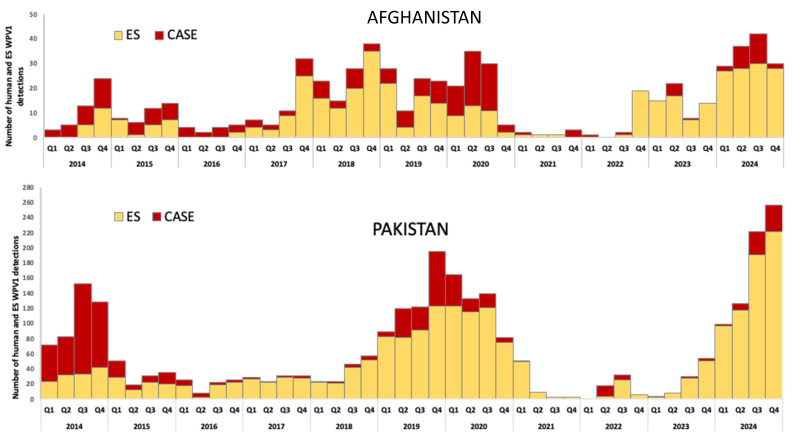
Number of wild poliovirus type 1 (WPV1) cases and number of environmental surveillance (ES) WPV1-positive samples by quarter for Afghanistan and Pakistan—2014–2024. Note: The number of ES sites substantially increased over this time period, which limits any time comparison of the number of ES detections.

During 2014–2024, the two countries reported 951 WPV1 cases, 732 (77%) of which were reported from Pakistan. The annual number of confirmed cases ranged from 5 in 2021 to 334 in 2014 (Figure 2). With 12 cases in 2023, a resurgence to 99 cases occurred in 2024; Afghanistan and Pakistan reported 25 and 74 WPV1 cases, respectively (Figure 2). WPV1 cases in Afghanistan in 2023 were detected only in the east region; in 2024, cases were also detected in the south region. In Pakistan, the first WPV1 detection outside of south KP occurred in October 2023 in Sindh province. In 2024, cases were additionally reported in Balochistan, Islamabad Capital Territory and Punjab.

**Figure 3:**
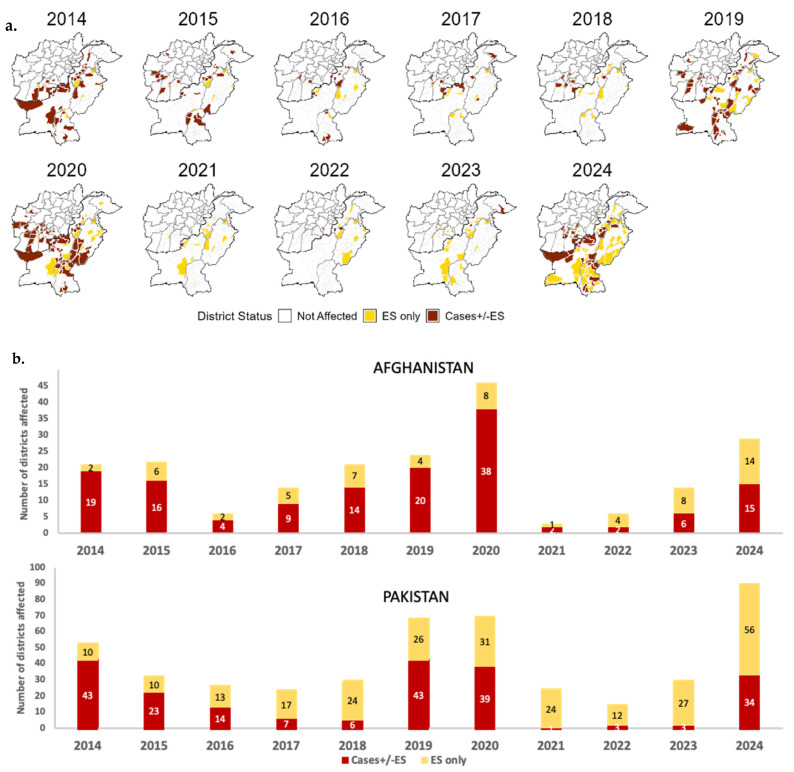
(**a**). Location of districts affected by wild poliovirus type 1 (WPV1) transmission confirmed by WPV1-positive cases and environmental surveillance (ES) samples. (**b**). Number of affected districts with WPV1-positive AFP cases or only WPV1-positive ES samples, by year, for Afghanistan and Pakistan—2014−2024. Note: The number of ES sites substantially increased over this time period, which limits any time comparison of the number of districts with ES detections.

Both Afghanistan and Pakistan have extensive ES networks to track the transmission of polioviruses, with 43 sites in Afghanistan and 147 sites in Pakistan in 2024, increases from 11 and 31 sites in 2014, respectively. The ES WPV1-positivity proportions of samples from Afghanistan for 2021, 2022, 2023 and 2024 were 1%, 2%, 13% and 23%, respectively, and for Pakistan they were 12%, 3%, 5% and 31%, respectively. Districts with WPV1-positive ES isolations during 2014–2024 are indicated in Figure 3a.

In 2014, 74 districts were affected by WPV1 transmission (cases and/or ES isolations) (21 in Afghanistan and 53 in Pakistan) (Figure 3a,b). The number of affected districts during 2014–2024 was lowest in 2022, with 21 affected districts (6 in Afghanistan, 15 in Pakistan) and highest in 2024 when 119 districts were affected (29 in Afghanistan, 90 in Pakistan) (Figure 3b).

During 2014–2024, 196 OPV SIAs were conducted in Afghanistan and Pakistan, including 90 national immunization days and 106 subnational immunization days in the highest risk areas (Figure 1). There also were numerous, small-scale outbreak response or mop-up campaigns. These SIAs utilized tOPV, bOPV and Sabin strain monovalent OPV type 1 (mOPV1). During January 2014–April 2016, tOPV was used for both SIAs and in RI. After the global switch from tOPV to bOPV in 2016, both countries implemented bOPV and/or mOPV1 campaigns until 2019 when first Pakistan and then Afghanistan programs detected cVDPV2 outbreaks and responded to cocirculation of WPV1 and cVDPV2 with mOPV2 and some tOPV SIAs.

The quality of the SIAs is assessed by post-campaign independent monitoring [12] and lot quality assurance sampling (LQAS) surveys [100]. In the latter, the “lots” assessed in Afghanistan are districts and in Pakistan they are Union Councils (UCs; subdistricts). Of the LQAS surveys conducted during 2014–2024, an average 25% of assessed lots in Afghanistan and 20% in Pakistan failed at the 90% quality threshold. The validity of the results of these assessments is variable: in Afghanistan, this is because of the reliance on local staff to conduct LQAS surveys. In Pakistan, this is because of intermittent evidence of “false finger-marking”, i.e., children not vaccinated although marked with silver nitrate markers on their fingernails as if they had been vaccinated [17]. Despite investments in improving administrative data management in both programs, the quality of data remains unreliable: although target population sizes are inflated to seek more resources, reported administrative coverage often exceeds 100% subnationally and nationally, even after including lower coverage in security-compromised parts of the countries. The two countries reported an estimated administrative average of 2% to 5% missed children after each campaign during the review period.

The two country programs have attempted to synchronize SIAs to ensure children living in border areas and those who move between the countries are reached during SIAs; however, this has been infrequent. During January 2021–December 2024, only 7 SIAs were fully synchronized among 28 SIAs conducted in Pakistan and 30 in Afghanistan.

##### Afghanistan Country-Specific Issues

Afghanistan has eight regions divided into 34 provinces with 400 districts. Program management in Afghanistan has not been smooth since the beginning of the Taliban overthrow of the government in 1996 and in areas under their control since 2001 (Human Rights Watch Backgrounder: available at https://www.hrw.org/legacy/backgrounder/asia/afghan-bck1023.pdf (accessed on 4 September 2025)). The ruling government in Kabul was intermittently committed to the polio eradication initiative depending on the person who represented the government and the person who was leading the Emergency Operation Centre (EOC) at the time. Nonetheless, twice prior to 2018, surveillance data suggested that all local transmission of WPV1 was interrupted; indigenous transmission apparently restarted due to population movements reintroducing clusters from Pakistan [101,102].

Between 2018 and 2023, three polio program team leads of UN agency offices were forced to leave the country because of disagreements on program implementation with government counterparts. The local Islamic State affiliate, ISIS Khorasan, had and has presence in the provinces of the east region with WPV1 circulation [45]. The Taliban has continuously controlled Helmand and Kandahar provinces in the south region and Farah province in the west region since 1996. With the geographic extent of control increasing from 2018, the Taliban took Kabul and deposed the government in August 2021. Pakistan intermittently attempted to restrict cross-border movements. During late 2023–early 2024, one million Afghans were forced to return from Pakistan [12]. Nonetheless, bidirectional cross-border movements continued.

In 2016, the GPEI supported the establishment of a national EOC within the Ministry of Public Health in Kabul and four regional EOCs. These institutions unfortunately lacked appropriate management structures and accountability mechanisms. The EOCs’ areas of influence before 2021 were restricted to those controlled by the internationally recognized government in Kabul. In addition, there was no consensus among the Ministry and GPEI partners on the specific functions of the EOC. Ministry authorities often directed the hiring of program staff who did not meet technical qualifications. After the Taliban assumed total control of the country, all EOC technical staff were replaced with personnel deemed acceptable by the Taliban public health representative, many without experience [103].

House-to-house (door-to-door) SIA implementation is a more effective means of reaching every targeted child when compared with implementation only at fixed sites at mosques, requiring caregivers to attend with their children [12,104]. Because provincial and local authorities could override central authorities’ policies, house-to-house vaccination implementation during SIAs could be highly variable. Provinces in the east and south regions intermittently stopped all SIAs for months at a time [84]. Bans on house-to-house SIA vaccination were imposed in many provinces in May 2018 and subsequently included periods of nationwide SIA bans on house-to-house delivery [90]. After the Taliban took national control in 2021, most restrictions on house-to-house vaccination were relaxed except in the south region [13]. During March 2022–July 2024, Afghanistan officials authorized an increasing extent of house-to-house SIAs implemented, essentially reaching nationwide (96–99% of districts) in June and July 2024 for the first time since early 2018, only to be banned nationwide again in August 2024 through today [12]. Kandahar city has not implemented house-to-house SIAs since the Taliban took national control and presents the biggest risk in sustaining WPV1 transmission in Afghanistan.

##### Pakistan Country-Specific Issues

Pakistan has four provinces (Balochistan, Khyber Pakhtunkhwa, Punjab and Sindh), two Government of Pakistan-administered areas (Azad-Jammu-Kashmir and Gilgit-Baltistan) and the Islamabad Capital Territory. Provinces are divided into administrative divisions (tehsils) that are further subdivided into a total of 159 districts and further subdivided into a total of 1710 UCs. Transmission of WPV1 from 2014 was most intense in the UCs that were security-compromised and difficult to access in districts in south KP and in the historical core reservoirs of Karachi, Peshawar and the Quetta block [105,106,107].

Security issues in Pakistan are not restricted to targeted hostilities against polio field workers. In KP and Balochistan provinces, several separatist movements and tribal enmity contribute to precarious security environments. As a means of improving community engagement in some high-risk areas, the program recruited women who were trusted by their local populations to repeatedly visit households to explain the benefits of and deliver OPV and provide other health information; they were known as community-based vaccination (CBV) workers. The CBV project was introduced in a few UCs in Karachi in late 2014 and progressively expanded until the end of 2018 [105,106,107]. Focused on the UCs that were security-compromised and hard-to-reach inside Karachi, Peshawar and Quetta, this intervention appeared instrumental in vaccinating more children but was also expensive and has since been reduced (National Emergency Operations Centre, Islamabad, Pakistan. National Emergency Action Plan for Polio Eradication 2020. Available at https://polioeradication.org/wp-content/uploads/2020/11/Pakistan-NEAP-2020.pdf (accessed on 4 September 2025)). Although restricted access due to insecurity remains in tribal areas of south KP, the major impediment in south KP districts is overall community resistance (boycotts) to repeated OPV SIAs without the delivery of other health or community services [46,108].

The national EOC (NEOC) and four provincial EOCs were established in 2015. Changes in management of the Pakistan polio program occurred after each change of government. August 2018–June 2024 was characterized by many political transitions and NEOC management turnover, resulting in suboptimal implementation of program activities and diminished oversight and accountability. In addition, from late 2019, the WHO country office, which leads in planning and overseeing SIA and surveillance operations, started to decrease the number of its international staff; this left national staff under greater influence of senior province and district government officials. Some senior officials perennially seemed to want to project programmatic progress externally. This tendency frequently deprioritized attention to key management and operational challenges that needed to be addressed. Following a national assembly vote of no confidence, the central government was disbanded and a transition government formed in April 2022, followed by two years of political instability [45]. After a new government was elected in 2024, a new NEOC coordinator was appointed, as was an influential Prime Minister Focal Point who resumed that position, having previously served in 2018.

Because isolations of WPV1 decreased nationally in 2021 and all 20 polio cases in 2022 were detected in south KP districts, the required focus on the historical poliovirus reservoirs was not maintained, leading to an insufficient number and quality of SIAs to keep WPV1 circulation restricted to south KP.

## 4. Progress Toward Ending Transmission of cVDPVs

### 4.1. Background

Sabin strain OPVs have a benefit of providing indirect immunization to some susceptible children in the community following shedding of poliovirus by vaccinees, particularly OPV2 in tOPV [47,109,110]. In under-vaccinated populations, however, prolonged community circulation of vaccine-related poliovirus can allow for reversion to VDPV (emergence) that has the neurovirulence and transmissibility biologically equivalent to WPV [111,112,113,114]. If transmission continues, cVDPV outbreaks of paralytic polio result.

Non-WPV poliovirus isolates from specimens are grouped into two categories based on the extent of divergence in the region coding capsid protein VP1 from the corresponding parental Sabin strain OPV: (1) vaccine-derived poliovirus (VDPVs), >1% divergent for types 1 and 3 and >0.6% divergent for type 2; or (2) vaccine-related poliovirus if less divergent [114,115]. VDPVs that are not immunodeficiency-associated [116] are further categorized as (1) cVDPV when there is evidence of person-to-person transmission [56]; or (2) ambiguous VDPV (aVDPV): unique poliovirus isolates from a person with AFP with no known immunodeficiency or from an ES sample, and no evidence of transmission of genetically related virus [114,115,117,118]. Although the initial VDPV criterion for all serotypes was >1% nucleotide divergence, experience during 2006–2010 in Nigeria [115,119,120] and the Democratic Republic of the Congo (DRC) [121] indicated neurovirulence (AFP) correlating with infection with type 2 poliovirus with 0.67–1.0% substitutions. Therefore, cVDPV2 case counts for Nigeria and DRC during 2005–2012 vary depending on data source, the original vs. revised criterion on the number of substitutions (some as few as 0.5% divergence), and may include cases of aVDPV2 infection [114,117,119,120,121,122,123,124,125,126].

#### 4.1.1. History of Use of Different OPV Presentations

Despite the relatively lower content in tOPV, OPV2 is the most effective of the three Sabin strain types and can interfere with seroconversion to PV types 1 and 3 (PV1, PV3) during the first doses of tOPV in a series; effective seroconversion to those two types occurs subsequently with later doses [47]. Because of the higher type-specific per-dose effectiveness against PV1 compared to tOPV [127,128], mOPV1 was introduced in India as the primary SIA vaccine in 2005 [129] to—theoretically—more effectively stop WPV1 transmission; it became the vaccine of choice for WPV1 outbreak response SIAs globally (Ad Hoc Advisory Committee for Polio Eradication, Geneva 22 April 2005. Available at https://polioeradication.org/wp-content/uploads/2024/05/2005042222_AACPE.pdf (accessed on 4 September 2025)). Following a surge in WPV3 cases in Uttar Pradesh, mOPV type 3 (mOPV3) briefly replaced mOPV1 in primary use there [129]. Ultimately, bOPV SIAs were introduced in 2010 to induce immunity against both WPV1 and 3 in India and other endemic and outbreak countries [122,130,131]. Notably, 16 cVDPV2 cases were reported in India during 2009–2010 [122]. The last detected cases of WPV3 in India occurred in 2010 (and globally, i.e., Nigeria and Pakistan, in 2012) [132]. Also beginning in 2010 in India, children of migrant workers who had been previously under-vaccinated in SIAs were specifically tracked, vaccinated and surveilled [8]. Subsequently, all WPV1 transmission was eliminated, with the last identified case occurring in March 2011 [8,133].

#### 4.1.2. The 2016 tOPV-bOPV Switch

To decrease the risk of cVDPV2 emergence, deliberation by the WHO Strategic Group of Experts on immunization (SAGE) during 2011–2016 resulted in the decision to stop all use of OPV2 [134,135,136,137,138,139,140,141,142]. Preparation for implementation began in 2014 for a global “switch” in 2016. Based on unpublished analyses from several modeling groups indicating widening immunity gaps to PV2 in areas with low RI tOPV coverage leading up to the switch, a series of tOPV SIAs were implemented during March 2015–April 2016 in some sub-Saharan African countries to increase population PV2 immunity [143,144]. Within a ~3-week window from mid-April to early May 2016, all OPV-using countries ceased use of tOPV, instead using bOPV in RI programs (and in SIAs); all high-risk OPV-using countries had already introduced ≥1 dose of injectable, inactivated poliovirus vaccine (IPV, containing types 1, 2 and 3 antigens) into the childhood RI schedule at 6 months of age [19]. Upon release by the WHO DG, mOPV2 was used in response campaigns to control cVDPV2 outbreaks. To mitigate mOPV2 seeding of emergences in outbreak response SIAs, nOPV2 was developed and ultimately used in SIAs beginning in March 2021 [31,145].

### 4.2. cVDPV Transmission, 2000–2024 (Figure 4a,b, 2014–2024)

In the 1990s, the GPEI markedly expanded PV surveillance and genomic sequencing analyses. Therefore, some cVDPV outbreaks prior to 2000 may have gone undetected (see Outbreaks Prior to 2014 below). However, such outbreaks may have been few: before 2020, natural immunizing infections with WPVs may have prevented wide circulation of Sabin-related poliovirus [146]. As GPEI-supported SIAs expanded and WPV exposures decreased, RI services in many areas remained weak, which increased the risks of cVDPV emergence [146]. Additional risk factors for cVDPV emergence and spread are high diarrheal disease incidence and high birth numbers [59].

**Figure 4:**
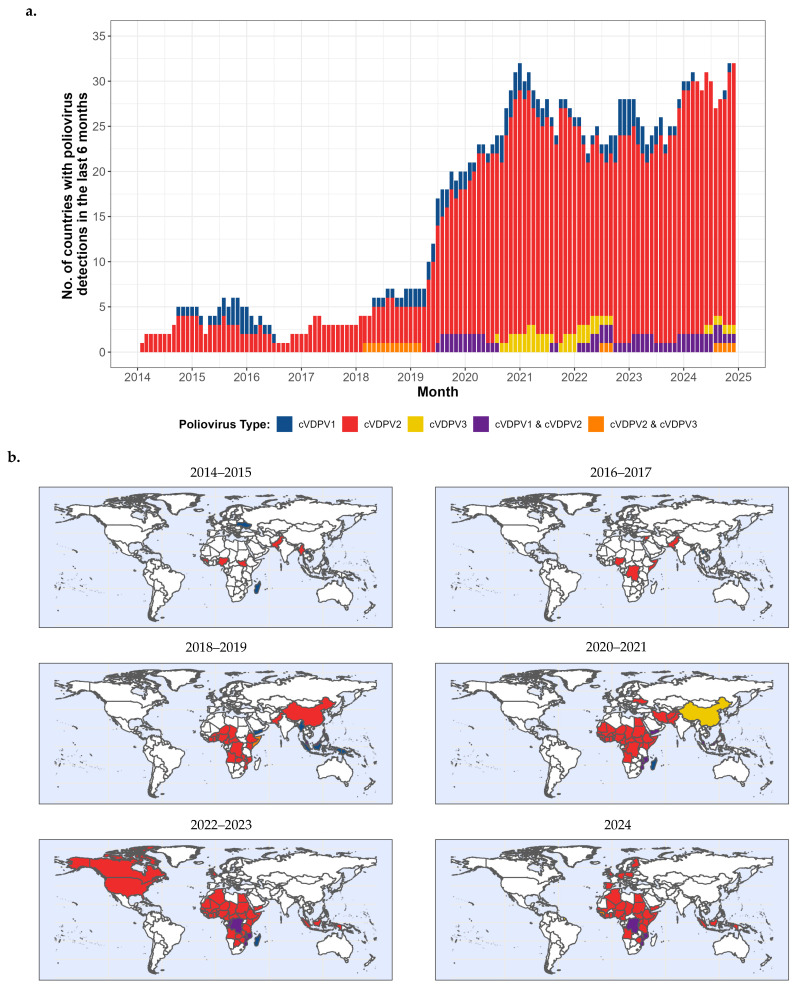
(**a**). Number of countries and areas with reported circulating vaccine-derived poliovirus (cVDPV) outbreaks identified by laboratory-confirmed AFP cases and/or environmental surveillance detection, by serotype (or types) and month, 2014–2014—worldwide. (**b**). Countries with cVDPV cases, by serotype or serotype cocirculation, 2014–2024—worldwide.

#### 4.2.1. Reported cVDPV1 and cVDPV3 Outbreaks, 2000–2024

The first reported cVDPV outbreak was cVDPV1 in Haiti and the Dominican Republic (Hispaniola) in 2000–2001 [112,147]. During 2000–2016, in countries with only RI services (i.e., without preventive SIAs), there were nine cVPDV1 outbreaks (including Hispaniola) with a range of 2–46 cases (median: 5; IQR: 2–11) [27,121,122,124,148,149,150,151]. During 2017–2024, there were 11 cVPDV1 outbreaks with a range of 1–265 cases (median: 6; IQR: 2.5–33.25) [26,34,152,153,154,155]. The most recent outbreaks were in Madagascar (2020–2023), DRC (2022–2024) and Mozambique (2022–2024). Prior to 2016, there were systematic, annual preventive SIAs implemented in DRC and West Africa to limit WPV1 outbreak risk and size that possibly also limited cVDPV1 outbreak risk and size [42,156,157]. There have been only six reported outbreaks of cVDPV3, with a range of 1–6 cases (median: 3.5), most recently in Israel (2022), French Guiana (2024) and Guinea (2024) [26,27,34,121,122,124,148,149,150,151,152,153,154,155].

#### 4.2.2. Reported cVDPV2 Outbreaks, 2000–2024 (Figure 4a,b, 2014–2024)

##### Outbreaks Prior to 2014

Retrospective sequence analysis revealed a large cVDPV2 outbreak during 1984–1993 in Egypt [158,159]. Prior to any SIA use of mOPVs, there had been two reported cVDPV2 outbreaks, in Madagascar (five cases in 2001–2002 and three in 2005) [160,161]. The relative frequency of cVDPV1 vs. cVDPV2 outbreaks over time has been affected by the OPV presentations used in RI and SIAs.

A single paralytic aVDPV2 case was identified in Nigeria in 2002 [117]. The first cVDPV2 case in Nigeria was detected in 2005, confirmed retrospectively; two unrelated paralytic aVDPV2 cases were also detected in 2005, both WPV1 coinfections (reported in WHO data as cVDPV2) [119,120]. Starting in March 2006, instead of tOPV, most SIAs in Nigeria regularly used mOPV1; mOPV3 was intermittently used during 2007–2009. Beginning in 2010, bOPV was predominantly used. During March 2005–June 2014, only 11 of 34 SIAs in northern Nigeria used tOPV; SIAs implemented during October 2007–May 2009 all excluded OPV2 [119]. Ultimately, Nigeria reported 403 “cVDPV2” cases during 2005–2011 from transmission of 7 cVDPV2 emergence groups and 16 individual aVDPV2 cases [120,124]; 11 of the 403 cases were WPV-coinfected [120]. Eleven more related cVDPV2 cases from a prominent 2006 emergence group [119] were reported during 2012–2013 [124,149]. Additionally, an outbreak of four cVPDV2 cases occurred in Nigeria in 2013 following importation of 2012 Chad cVDPV2 emergence [124].

During 2006–2013, there were 228 cVDPV2 cases outside of Nigeria. Niger reported cases due to cVDPV2 first detected in Nigeria. Countries reporting outbreaks included Afghanistan, DRC (multiple emergences), Chad (spread into Cameroon and Nigeria), China, India, Pakistan, Somalia (spread into Ethiopia and Kenya) and Yemen [115,121,122,124,149].

##### cVDPV2 Outbreaks, 2014–2024

Prior to the 2016 switch, there were new outbreaks in Guinea (8 cases, 2014–2015), South Sudan (2 cases, 2014) and Myanmar (2 cases, 2015) and residual transmission of prior outbreaks in Nigeria (32 cases, 2014–2016) and Pakistan (25 cases, 2014–2016) [27,150,151]. Continued cVDPV2 transmission in Borno, northeast Nigeria, that had spread from Chad went undetected in 2015 but was again detected in an ES sample taken in March 2016; genetically linked cVDPV2 was isolated from a healthy child’s stool sample taken in July [69,162]. Another ES isolate related to the predominant outbreak emergence group from 2006 to 2013 was found in a sample taken in March 2016 in another state. For both March ES samples, laboratory results were only available in late April 2016 [69,151,162]. In late 2016, immediately following the switch, new independent emergences resulted in one cVDPV2 case and several aVDPV paralytic cases that occurred in Sokoto state, northwest Nigeria [27].

Post-switch, a series of outbreaks were identified, reaching a peak of 1082 cases in 2020 before annually receding into hundreds of cases; the geographic scope is indicated in Figure 4b. In Pakistan, five independent cVDVP2 emergences were identified in 2019 [154]. There apparently was inadvertent use of inappropriately retained tOPV in the initially affected areas because there were also multiple isolations of Sabin strain vaccine-related PV2, although investigations found no evidence of such tOPV use [25,154]. New emergences subsequently occurred from seeding by low-quality mOPV2 response SIAs. Transmission spread to Afghanistan in 2020 where seeding of new emergences also occurred [154]. No cVDPV2 transmission was detected in either country after June 2021.

Some early cVDPV2 outbreaks in 2017 were related to emergence occurring years before the switch but were discovered after the switch, as in DRC and Somalia [27,152]. Most outbreaks resulted from emergences occurring peri- or post-switch. Substantially long and large outbreaks during 2017–2024 occurred in Algeria (2022–2024), Angola (2019–2020, 2024), Chad (2019–2020, 2022–2024), DRC (2017–2024), Ethiopia (2019–2024), Somalia (2017–2024) and Yemen (2021–2024); the post-switch outbreaks in DRC (2017–2024) had some international spread not only into Angola but also Burundi, Tanzania and Zambia [26]. Some outbreaks were smaller/more quickly controlled such as the indigenous outbreak involving the Philippines and Malaysia (2019–2020) and in Tajikistan (with extension into Ukraine, 2021) of cVPDV2 of Pakistani origin.

As of 27 March 2025, there were 3733 paralytic cVDPV2 cases identified during 2014–2024 (3664 during 2017–2024, post-switch) in 45 countries (Figure 4a,b); of the total 4361 cVPDV2 cases reported during 2000–2024, 86% had onset during 2014–2024 (84% during 2017–2024). An additional 16 countries have identified transmission of cVDPV2 only by ES. A single confirmed cVDPV2 case in the United States in 2022 was related to cVDPV2 isolated by ES sampling in New York State and to vaccine-related PV in ES samples taken in New York State, Israel, the United Kingdom and Canada [155,163,164,165,166]. In 2024, during Israel’s war against Hamas in Gaza, Occupied Palestinian Territories, cVDPV2 transmission of an emergence group originating in Egypt was identified with a confirmed case and ES isolations [26,167].

Because of suboptimal response SIAs, new emergences were generated in some countries following response SIAs with mOPV2 (n = 51) or nOPV2 (n = 19, reported during 2021–2024, as of 27 March 2025). A substantial number of cVDPV2 cases reported after the switch (2017–2024) resulted from cVDPV2 emergences that were of Nigerian origin (1381/3664, 38%) in outbreaks that swept west to Senegal and east to Sudan and South Sudan. Just two emergence groups from Nigeria of the 15 emerging post-switch there caused 665 (48%) of the 1381 cases in 10 countries outside Nigeria. Nine other countries identified cVDPV2 transmission of Nigerian origin by ES sampling only. Five were western European countries that isolated the predominant cVDPV2 of Nigerian origin from ES samples taken during 29 September–29 December 2024 [168]; in three countries, isolations continued from samples taken in 20 January–28 July 2025 (https://polioeradication.org/circulating-vaccine-derived-poliovirus-count/ (accessed on 4 September 2025)).

## 5. Addressing Challenges to Polio Eradication

### 5.1. Overview

The programmatic prolongation of the GPEI since 2014 has resulted from the difficulties in addressing numerous challenges, some of which were evident soon after the GPEI was established 37 years ago. These challenges are similarly affecting efforts to stop cVPDV2 transmission. We review recurring and other challenges that country public health authorities and GPEI leadership currently face (see Table 1 for summary). Although individual challenges may be effectively mitigated, these challenges collectively threaten the success of polio eradication. None of the listed mitigations to address challenges are panaceas.

Until 2025, the primary contemporary challenge to the success of the GPEI was poor activity implementation secondary to low government ownership of the activities and outcomes. However, decreased funding of the GPEI by international development country donors in early 2025 has become an acute challenge that could lead to all progress to date being reversed if needed operations are not implemented; field staff have already been reduced in all GPEI-supported countries. The Kingdom of Saudi Arabia has recently joined the GPEI partnership [169]. Other donors need be sought, including other high-income countries and high-net-worth individuals, increased funding of national activities by affected lower-middle-income countries must be sought.

### 5.2. Recurring Challenges

#### 5.2.1. National Engagement

A major recurring challenge is limited country ownership of eradication program activities, often accentuated during and after changes in government [45,46]. This is manifested by low engagement in operational accountability, poor community involvement, limited logistical support, and weak supervision. Imprudent, biased misinterpretations of data allow decisions to be made based on political expediency. Limited ownership enables corruption to be overlooked at many levels and sustains field improprieties like “false finger-marking”. When the Nigeria program established national and state EOCs in 2012, the extent of program ownership by federal, state and local government authorities grew. This did not persist after WPV-free certification of the WHO African Region as funding priorities are now set at the lowest level [46]. Discovered in Nigeria in 2023 and 2024, “false finger-marking” in areas that are not security-compromised indicates limited field team accountability and misguided vaccinators’ attitudes towards vaccine safety.

##### Mitigations

Regaining or retaining national engagement requires persistent, coordinated advocacy by high-level representatives of GPEI partner agencies, other international organizations and other internal and external stakeholders. Logistical support may require aircraft, off-road vehicles, motorcycles, watercraft and fuel. Engagement challenges may have to be met with new placement of international GPEI staff at national and subnational levels. When government leadership changes hands, changes in priorities need to be anticipated and GPEI advocacy needs to be bold. In Pakistan, the new government’s engagement and new EOC management in 2024 are conducive to improved operations.

#### 5.2.2. Limited Security and Access

Before and since the GPEI began, there have been impediments to the immunization of children residing in areas with active conflict or that are otherwise security-compromised [170]. In Pakistan, the threat of violence directed against workers and/or against their security details remains. Al Shabab-held areas of South-Central Somalia and Houthi-held north Yemen have had severely restricted vaccination activities [45,46]. Insecurity also results from armed criminality, such as road banditry, kidnapping or fatalities of non-residents entering areas held by armed extortionist groups, functionally equivalent to an insurgency [171]. This has been occurring in a corridor across several northwest states in Nigeria, the reservoir of established cVDPV2 transmission since 2020, widening into north central states.

##### Mitigations

Addressing security-compromised access challenges requires implementing variable, innovative efforts by public health authorities [77,78,172]. This includes security personnel accompanying immunization teams or collaboration with humanitarian organizations in the countries to negotiate safe access, even if for limited time periods. Some insurgent-held areas in South-Central Somalia are allowing entry for SIAs through negotiation by humanitarian organizations offering other health services [173]. From civil wars in Latin America countries in the late 1980s up to the war in Gaza in 2024, ceasefires have been successfully negotiated to allow for SIA implementation [78,167,172,174,175]. In some uncontrollably insecure areas in Nigeria, large-scale action by state government authorities may be needed to protect civil society and shrink security-compromised areas [171].

#### 5.2.3. Other Access Impediments

First, marginalized, minority populations may not be included in the provision of health services through neglect. There are marginalized populations and underserved areas in many countries that require specific planning of active interventions to reach them with immunization services, such as families of fishermen or pastoralists [176]. For example, pastoralists were implicated in poliovirus spread within Nigeria and Lake Chad countries. These pastoralists in Nigeria were predominantly ethnic Fulani; temporary pastoralists’ camps and even settled Fulani communities were generally overlooked in microplans for SIA implementation before 2006, and this persisted in some locales until 2012 [177,178,179]. Before the simultaneous cVDPV1 and cVDPV2 outbreaks during 2019–2020 involving indigent, minority populations inhabiting and transiting between the Philippines and Malaysia, the government of Malaysia did not provide immunization services to non-citizens [180]. Second, another access problem is logistically hard-to-reach populations, such as underserved riverine communities in DRC or interior areas in Madagascar. Third, another major “access” impediment is low household and community acceptance of OPV SIAs—including outright boycotts—because they are resistant to “vaccination against one disease only, so often” without the community having access to other essential health services or community services, such as electricity or clean water. Boycotts for these reasons are particularly a problem in south KP districts in Pakistan [45,46,181,182].

##### Mitigations

The least served subpopulations need the most consideration. Full logistical support is needed to reach hard-to-reach geographies, whether riverine or remote areas without roads. Implementing multi-antigen vaccination efforts can address some community concerns about a vaccination focus “only on polio”. Examples of adding other interventions include rehydration electrolyte packets, hand soap or anthelminthics. On a larger scale, offering “health camps” including health services for children of all ages has been a successful means of vaccinating children in resistant communities in areas in Nigeria during WPV transmission [183] and in Pakistan [45,181,184]. Organizing more camps and promoting efforts to increase the provision of clean water, electricity or other services could be considered through multisector or bilateral collaborations that might spur resource investment by governments.

### 5.3. Selected Groups of Other Major Challenges

#### 5.3.1. Residual Effects of COVID-19 Pandemic

The pandemic’s effect on the quality of poliovirus surveillance and SIAs created substantial impediments to polio eradication progress during 2020–2021 [36,38]. Planned SIAs were postponed during March–June 2020 and subsequently rescheduled SIAs were often deferred [36]. Public health workers and available financial resources were diverted to COVID-19 surveillance and community interventions, and then to the introduction of COVID-19 vaccination. Outbreak control SIAs for cVPDV2 conducted in 2021 generally were delayed and of low quality in sub-Saharan African countries. Since recovery from the acute phase of the pandemic, national authorities have frequently cited other public health threats as higher priorities to address rather than promptly implementing poliovirus outbreak response SIAs; when implemented, quality is frequently substandard, even recently.

There was a major decrease in RI coverage in 2020 globally, worsening in 2021, due to a decrease in caretaker visits to immunization clinics, restricted clinic operations and a decline in outreach activities, therefore also lowering IPV coverage [185,186]. Gavi, the Vaccine Alliance, began offering support in 2016 for the identification and vaccination of “zero-dose” (i.e., totally unvaccinated) children in concentrated areas, primarily with periodic intensification of RI services/outreach. However, activities were reduced during and after the acute phase of the pandemic [187]. In addition, the COVID-19 vaccination effort was unfortunately accompanied by the rapid spread of misinformation across social media and widely shared within countries—primarily from high-income countries—about the safety and efficacy of not only COVID-19 vaccines but also RI vaccines.

##### Mitigations

With a small rebound of RI coverage in many countries in 2022 and 2023 in children 12–23 months of age, the WHO, UNICEF and Gavi organized an effort to immunize missed children in new and older cohorts (<5 years of age) during 2023–2025 [188,189]. The “Big Catch-Up” with RI antigens did not always include bOPV but included IPV (resources available at TechNet https://www.technet-21.org/en/topics/programme-management/the-big-catch-up#:~:text=Launched%20in%20April%202023%2C%20the,(MEL)%20forms%20and%20tools (accessed on 4 September 2025)). Countering vaccine misinformation requires persuasive social media messaging, increased community engagement and renewed recruitment of community opinion leaders [45]. Global RI coverage was unchanged in 2024 [190].

#### 5.3.2. Outbreak Response Capacity

With major exceptions in Nigeria and Somalia, cVDPV2 outbreaks detected in 2016–2018 that emerged pre-switch, peri-switch or seeded following mOPV2 use were more effectively controlled than outbreaks in subsequent years [191,192]. Early responses meeting WHO poliovirus outbreak standard operating procedures’ timelines [56] were few; nonetheless, the more promptly response SIAs were initiated, the more rapidly transmission was interrupted [191,193]. Limited supply of filled vials of mOPV2 and then of nOPV2 constrained the appropriate scope and timing of some response SIAs. Adequate nOPV2 supply is no longer a substantial issue; however, there continue to be delays in initiating prompt response SIAs after laboratory confirmation. International poliovirus transmission remains a PHEIC but sustaining that status has not accelerated the promptness of post-pandemic outbreak response activities. Delaying implementation of outbreak response SIAs to organize coordinated multi-country campaigns is counterproductive to timeliness but has occurred. Given the necessity to effectively interrupt ongoing transmission, it is illogical to invest resources in initiating a first or second response round six months to a year after a single cVDPV isolation. The insufficient quality of SIAs with mOPV2 and nOPV2 have not only failed to stop some active cVDPV2 transmission in countries of the WHO African Region but has also seeded new emergences into 2024 [26] (https://polioeradication.org/circulating-vaccine-derived-poliovirus-count/ (accessed on 4 September 2025)).

##### Mitigations

Outbreak responses urgently need GPEI partners and affected country authorities to resume an emergency footing to succeed. The time from outbreak confirmation to preparation for suitable response SIA implementation requires close GPEI monitoring and advocacy intervention as appropriate. WHO Regional Directors, the DG or other highly regarded individuals may need to aggressively advocate country prioritization of prompt and effective responses with ministers of health and/or heads of state. GPEI partners need to ensure prompt technical assistance and operational funds. The scope of response SIAs needs to be appropriately sized to be implemented promptly. Utilizing direct detection laboratory methods and increasing the number of accredited sequencing laboratories in the African region will ultimately assist in more rapid outbreak confirmation [194,195,196,197]. More importantly, there are still major delays in specimen handling and transportation and PV isolate transfer for characterization; these shipments can be more closely tracked and delays more promptly addressed. Locating a depot of nOPV2 doses on the African continent in a location with well-connected airline services may shorten response times by shortening the delivery intervals. Increasing coverage of first and second IPV doses, including through SIAs [127,198,199,200,201,202], can decrease the number of cVDPV2 cases in affected countries. SAGE has recommended IPV inclusion in response SIAs as early as feasible [203,204].

#### 5.3.3. Strategic Planning and Decision-Making

Major progress toward eradication since 1988 was the result of clear policies, standardized interventions, surveillance performance indicator monitoring, program persistence applied globally and many other factors that provided valuable lessons [205]. The governance and oversight structure of the GPEI has changed substantially over time [42]. The WHO has been the guiding agency since GPEI inception, with close partner agency consultation and collaboration. In the last decade, there has been increasing focus within the WHO on transitioning to a “polio-free” world, with an emphasis on overall health system strengthening. This was accompanied by decreases in WHO and UNICEF country GPEI staff numbers who were engaged in many activities to strengthen overall health systems. Additionally, the effect of decreasing GPEI funding to some polio-free countries with weak RI and surveillance led to a need to recommit substantial resources when outbreaks occurred, such as in DRC during 2017–2024, Nigeria during 2016–2024, and recently Guinea and other West African outbreak countries once again [26]. Partners anticipated that GPEI fund-recipient lower-middle-income countries would themselves invest in strengthening essential health services as external GPEI funds were decreasing; the reality has been little self-investment. The risks and severity of cVDPV outbreaks were often under-appreciated by some WHO staff [46]. The GPEI partnership often became risk-tolerant rather than systematically evaluating risks that could have been mitigated at a much lower cost than when the risk actualized and acting as needed. An example is the large and lengthy cVDPV1 outbreak in DRC, anticipated because of very low RI coverage in many subpopulations [42]. Because of the number of outbreaks in recent years, GPEI management has unfortunately needed to spend more discussion time on budgeting for outbreak responses than on strategic issues.

During 2010–2014, preventive SIAs were annually implemented following WPV outbreaks cascading west and east from Nigeria during 2003–2009 to decrease the likelihood and size of subsequent WPV outbreaks [156,206]. The number of countries conducting preventive SIAs has decreased since 2015; even with pre-switch tOPV SIAs, the decline has continued, and essentially no preventive SIAs have been implemented since 2022 [42,207]. Where RI coverage is very low (e.g., <50%) in subnational areas and communities, the risk of cVDPV1 outbreaks has risen over time [207,208].

##### Mitigations

GPEI leadership needs to continuously re-evaluate risks based on the output of several modeling groups when considering adopting or modifying risk mitigation measures. This includes taking executive actions for mitigating the underlying reasons for low-quality outbreak SIAs and ensuring improvements. Strategic planning needs to be at the forefront of GPEI management discussions, and balanced consideration of high-risk contingencies and regional autonomy with global-level policies and fund-raising/allocation is necessary. Priorities need to be set about the use of limited cVPDV2 outbreak resources (funds and nOPV2) so that newly identified outbreaks are quickly provided with the means of promptly stopping them. The *GPEI Strategic Plan 2022–2026* (budget of USD 5.1B) has been extended to 2029, with a total budget of USD 6.88B [209]. As the IMB recommended, extension of the Plan may permit implementing preventive bOPV SIAs during 2025–2029 [45] in areas of countries with low national/subnational RI coverage, if funds allow. Lastly, there need to be reliable signs of reaching GPEI goals before transitioning resources.

## 6. Discussion

All GPEI partners and stakeholders recognize that the arc of progress toward WPV1 polio eradication since 2014 has been lengthened beyond expectations, and that stopping ongoing cVPDV2 transmission since 2017 is an additional goal with limited progress. The WHO Executive Board in February 2025 stated that emergency measures are urgently needed to stop continued WPV1 and cVDVP2 transmission and advocated that appropriate geopolitical decisions be urgently made [210]. To end all PV transmission in the near future, urgent and focused actions are needed to address the many ongoing and recent challenges. The problems of accessing children in security-compromised areas are not new and have heavily contributed to that lengthened arc of progress and cVDPV2 transmission; effectively mitigating those problems is clearly not easy [45]. However, low-quality SIAs in secure areas perpetuate transmission and international spread. A major challenge added in 2025 is an acute crisis in funding the GPEI, which requires swift global mitigation. Field staff are being released, and cash flow has been hindered.

Tracking progress toward eradication by trends in WPV1 case counts can lead to overly optimistic planning of program activities. ES sampling has added immensely to poliovirus surveillance sensitivity and has been expanded many-fold [54,211,212,213]. Nonetheless, the reach of ES sampling into some areas is limited by its implementation requirements [52,53]. To end all WPV1 transmission, the GPEI and country programs need to (1) account for cyclical (biennial) WPV1 transmission patterns, (2) monitor the areas where circulation is newly detected by ES sampling to guide prompt programmatic activities and (3) avoid premature scaling-back of SIAs. Continuing to rigorously implement SIAs outside of the active reservoirs will mitigate the potential for outbreaks following population movements into other high-risk areas.

The decrease in WPV1 cases in Afghanistan and Pakistan in 2021 and the cessation of 2019–2021 cVDPV2 transmission followed 2020–2021 pandemic precautions. Resumption of a schedule of SIAs during 2022–2023 was not sufficiently effective to prevent WPV1 spread across each country in 2023 and 2024. WPV1 transmission has continued in both into 2025 (https://polioeradication.org/wild-poliovirus-count/ (accessed on 4 September 2025)). Persistent indigenous WPV1 transmission can be interrupted in Afghanistan and Pakistan when the current resurgence is again reduced to the 2021 reservoir districts in each country and simultaneously by better penetrating vaccination efforts in those districts. The underlying reasons for non-vaccination in Afghanistan and Pakistan communities need to be better addressed by as many innovative tactics as possible. In Afghanistan, without resumption of national house-to-house vaccination, program reach will remain suboptimal. Combined with very weak RI services, continued SIA under-vaccination of young children limits the likelihood of interrupting WPV1 in the immediate future. House-to-house vaccination SIA implementation can only be resumed when the current authorities reevaluate. To stop all transmission in Pakistan, GPEI partner agencies would be best served if their most innovative and skilled staff are critically placed in their country offices. Ending WPV1 transmission by the end of 2026 can only be anticipated if all efforts can be implemented and sustained throughout 2025–2026 in both countries simultaneously.

Ending all cVDPV2 transmission is a more complex issue with the absence of intestinal immunization with OPV2 in RI. What was projected to be a few years of responding to cVDPV2 outbreaks after the switch has extended to nearly a decade. Persistent or established transmission continues in six countries currently: Algeria, Chad, Ethiopia, Nigeria, Somalia and Yemen. In alignment with the GPEI’s stated strategic goal, eventual discontinuation of all live, attenuated poliovirus vaccines remains a key milestone in attaining a polio-free world [44]. Global analyses that relied on inferences from non-polio AFP case dose histories, many unpublished, suggested that pre-switch PV2 immunity in sub-Saharan Africa was high, particularly in most of Nigeria [144,214]. Voorman et al. in 2023 used other methods [207]. Those analyses that relied on non-polio AFP case dose histories apparently masked substantial residual pre-switch PV2 susceptibility in key subpopulations of many countries. Caretaker recall OPV dose histories taken during investigations of children with non-polio AFP have been generally useful in assessing poliovirus population immunity in Afghanistan, Pakistan and globally [37,144,214,215]. However, relying on these histories for many African countries, where such histories may be more casually obtained than elsewhere and/or based on geographically skewed AFP surveillance [119,156], perhaps overestimates PV immunity for all serotypes [144,214].

The emphasis in Nigeria on implementing OPV SIAs without PV2 in the many years preceding the switch—to enhance immunity against PV1—resulted in a high proportion of those cohorts being missed by both weak RI services and the relatively few tOPV SIAs [68]. The outcome in Nigeria paralleled the experience in India, where temporary preferential use of serotype-unbalanced OPV preparations in SIAs resulted in a surge in WPV3 transmission and cVDPV2 emergence and spread. WPV1 transmission in India likely ended as a result of operationally reaching more disenfranchised subpopulations [8] rather than implementing SIAs with higher per-dose OPV type 1 effectiveness, given repeated SIAs [42]. In Nigeria, major preferential use of serotype-unbalanced OPV preparations in SIAs prior to the switch allowed for profound residual PV2 susceptibility [45]. The benefit of marginally higher per-dose bOPV type 1 effectiveness vs. tOPV was not worth the loss in pre-switch PV2 population immunity [10,74,75,76,79,216].

An external evaluation of the tOPV-bOPV switch indicated that implementation was structurally strong but strategically a failure, primarily because of an inability “…to close out outbreaks and stop cVDPV2 transmission. Outbreak response scope, timing and quality have been insufficient, resulting in increased scope and magnitude of cVDPV2 transmission over time…” [25]. The reviewers listed several critical issues that contributed to that strategic failure, including not meeting all switch prerequisites such as failing to introduce IPV in all countries only using OPV in RI before the switch. IPV supply limitations prevented many lower-risk countries from introducing IPV for up to two years [25,217]. Because of substantial gaps in poliovirus surveillance sensitivity, particularly in security-compromised areas, outbreaks of cVDPV2 that were seeded well before pre-switch tOPV SIAs in DRC, Nigeria and Somalia were only detected post-switch [25,27,152]. Pre-switch tOPV SIAs in high-risk countries were not implemented with enhanced efforts to reach populations in subnational geographies with very low RI coverage and prior low-quality SIAs. The underlying strategic failures of the switch were assuming adequate subnational poliovirus surveillance in high-risk countries regarding ongoing transmission and underestimating the number and size of communities with very low PV2 immunity at the time of the switch [143,218]. These lessons are critical to note when preparing for ultimate bOPV cessation.

International spread has been facilitated by (1) weak PV surveillance, (2) delays in cVDPV2 detection due to delayed investigations and/or sample shipments within countries and to laboratories and (3) delays in implementing SIA responses after laboratory confirmation. Ongoing spread after cVDPV2 importation into countries will only be limited with prompt, adequate outbreak responses. A recent analysis points to the increasing need for SIA quality with decreasing population intestinal immunity over time [219]. Suboptimal outbreak response scope and quality have allowed for the seeding of new cVDPV2 emergences. Although the use of nOPV2 for outbreak response has a lower risk of seeding new emergences than Sabin strain mOPV2 [220], the extent of that benefit is diminished when low-quality SIAs are implemented. Low-quality nOPV2 SIAs allow for prolonged circulation of vaccine-related PV, increasing the risk of subsequent non-polio enterovirus recombinations that remove key genomic modifications to reduce reversion. Subsequent reversion of attenuating sites results in nOPV-cVDPV2 (cVDPV2-n) emergence and spread [26,221,222].

The stopping of all cVDPV2 transmission globally will take a concerted effort by all stakeholders—GPEI partners, national ministries of health, subnational health officers and outbreak response authorities—to act quickly and improve the effectiveness of response efforts: (1) prompt outbreak responses as befitting a PHEIC need to be much improved from the period 2021–2024; (2) the SIA scope must include geographic areas of presumptive or threatened transmission but not be so large as to tax or deplete resources or delay implementation; (3) more effective penetration/quality of SIAs in secure areas is needed; (4) more effective negotiation for vaccination access in security-compromised areas or other innovations is required; and (5) implementation of at least three nOPV2 response SIAs in new outbreak areas is needed [25,223], not in the current standard operating procedures [56]. Nigeria poses the highest risk of continuing to internationally spread cVDPV2, followed by Chad and by Ethiopia, where civil conflict continues. That risk includes spread into countries of other WHO regions where young children have no PV2 intestinal immunity and IPV coverage is suboptimal. Extensive tactical changes need to be made by the Nigeria program to prevent its established transmission reservoir from repeatedly spawning international spread and to end all transmission. Narrowing the IMB recommendations of implementing preventive nOPV2 SIAs [46], we propose an encircling corridor of nOPV2 SIAs in areas/countries around the reservoir.

There remains a high risk of not stopping persistent cVDPV2 transmission by the end of 2026 in Algeria, Chad, Ethiopia, Nigeria, Somalia and Yemen, all reporting cVDPV2 isolations in 2025 (https://polioeradication.org/circulating-vaccine-derived-poliovirus-count/ (accessed on 4 September 2025)). Containing ongoing transmission in 2025 within the security-compromised reservoirs of Nigeria and Somalia and preventing wider circulation are imperative to preserve resources and direct them to these reservoirs. It may be necessary to temporarily introduce nOPV2 into the RI schedule in Nigeria or in the highest-risk areas [223]; however, that risks nOPV2-related PV transmission to other countries and its consequences. Yemen will remain an unchecked reservoir that risks serving as a source for international spread until anti-government authorities can be persuaded to implement outbreak response SIAs.

Polio eradication has been modeled as vastly more cost-effective than “permanent control”, even with delays in reaching the eradication goal [224,225,226,227]. Depending on the level of “control”, the number of paralytic WPV1 cases occurring annually could return to >100,000 and increase the risk of international spread into high- and upper-middle-income countries. Over 20 million children have been spared of paralysis since 1988 due to polio eradication SIAs and strengthened RI services [41]. To reach WPV1 and cVDPV2 eradication in the near future, GPEI partners need to mobilize the resources needed both urgently and over time to address the challenges to fully interrupting transmission everywhere and very effectively regain the prior momentum of the eradication initiative.

## 7. Conclusions

Interrupting all WPV1 transmission will depend on the sustained implementation of highly effective activities in both Afghanistan and Pakistan simultaneously. In Afghanistan, without resumption of national house-to-house vaccination, program reach will remain suboptimal. In Pakistan, the new government’s engagement and revised NEOC leadership offer promising opportunities to improve operations. Even if improvements are effectively pursued in both countries throughout 2025–2026, interruption of WPV1 transmission in 2026 remains at high risk. A key barrier to interrupting both WPV1 and cVDPV2 transmission is limited ownership/engagement. Insecurity continues to constrain access to certain subpopulations, and community resistance presents an additional obstacle to vaccination efforts. Misinformation spread across social media has exacerbated vaccine hesitancy in many communities. Addressing these challenges will require adaptive approaches that enhance access and community trust. The changes in public health priorities and practices that have lingered after the acute phase of the COVID-19 pandemic have contributed to longer-term delays in cVDPV2 outbreak response campaigns. The acute funding shortfall starting in 2025 requires renewed and expanded global support to avoid reversal of GPEI progress to date. To resume progress toward eradication, it will be important for GPEI leadership to continue addressing emerging challenges with urgency and coordination.

## Figures and Tables

**Table 1:** Major challenges faced by the Global Polio Eradication Initiative (GPEI) and potential mitigations—2025.

Category	Specific Challenges	Potential Mitigation Measures
Funding Shortfall in 2025	• Unexpected donor country funding decreases, with immediate impact on staffing	⬪ Recruit more countries and high-net-worth individuals to participate as donors in global funding ⬪ Seek increased internal funding of operations by lower-middle-income countries with poliovirus transmission
Limited National Engagement	• Low operational accountability and limited logistic support • “False finger-marking”	⬪ Coordinate advocacy by high-level representatives of GPEI partner agencies, other international organizations and other stakeholders ⬪ Place more international GPEI staff at national and subnational levels to enhance accountability
Limited Security and Access	• Civil war and insurgency	⬪ Negotiate “days of tranquility” (temporary ceasefire) ⬪ Collaborate with humanitarian organizations to negotiate safe access ⬪ Implement variable, innovative efforts relevant to security level
	• Targeted violence	⬪ Have security personnel accompany immunization teams
	• Armed criminality, kidnapping	⬪ Seek large-scale action by state governments
Other Access Impediments	• Marginalized subpopulations	⬪ Seek subpopulation engagement in campaign microplanning
	• Hard-to-reach riverine and remote communities	⬪ Seek necessary national logistical support: aircraft, off-road vehicles, motorcycles, watercraft and fuel
	• Low community acceptance of only oral poliovirus vaccine	⬪ Provide multiantigen outreach vaccination ⬪ Provide “health camps” including health services for children of all ages ⬪ Provide other services, e.g., sanitation and clean water
Residual Effects of COVID Pandemic	• Other health threats are higher priorities	⬪ Coordinate high-level advocacy for promptness of outbreak responses
	• Decreased routine immunization coverage	⬪ Increase IPV delivery with periodic intensification of routine immunization and outreach services
	• Vaccine misinformation and “false finger-marking”	⬪ Increase community engagement and recruit high-level and community opinion leaders equipped with persuasive messaging
Outbreak Response Capacity	• Delayed and suboptimal quality outbreak response SIAs and “false finger-marking”	⬪ GPEI partners and affected countries resume an emergency footing ⬪ Rapidly provide funds, human resources and vaccine ⬪ Coordinate high-level advocacy for promptness ⬪ Enhance supervision for SIA quality and accountability
	• Delayed specimen and isolate handling and shipment	⬪ Track shipping of specimens subnationally and internationally and intervene as needed
Strategic Planning	• Premature focus on funding transition to health services *	⬪ With appropriate integration of services in the interim, delay transitioning resources until there are reliable signs of reaching GPEI goals
	• Prioritizing and monitoring rapid outbreak responses	⬪ Ensure that resources are urgently directed to new outbreaks ⬪ Systematically evaluate risks and mitigate them before a risk becomes actualized

* Emphasized enhanced delivery of RI services vs. maintaining number and quality of SIAs until PV transmission ends.

## Data Availability

Not applicable.

## Dedication

In fond memory of John Sever, the dedicated motivator of Rotary International members to commit to supporting polio eradication and an integral person in sparking and expanding GPEI, and of Aidan O’Leary, Director of the WHO Polio Eradication Division during 2021–2024.

## Abbreviations and Initialisms

The following abbreviations are used in this manuscript:

AFP	Acute Flaccid Paralysis
aVDPV	Ambiguous Vaccine-Derived Poliovirus
aVDPV2	Ambiguous Vaccine-Derived Poliovirus type 2
bOPV	Bivalent Oral Poliovirus Vaccine (Sabin strain types 1 and 3)
CBV	Community-Based Vaccination (Pakistan)
COVID-19	Coronavirus Disease 2019 due to SARS-CoV-2
cVDPV	Circulating Vaccine-Derived Poliovirus
cVDPV1	Circulating Vaccine-Derived Poliovirus type 1
cVDPV2	Circulating Vaccine-Derived Poliovirus type 2
cVDPV3	Circulating Vaccine-Derived Poliovirus type 3
cVPDV2-n	Circulating Vaccine-Derived Poliovirus type 2 emergence from nOPV2
DG	Director-General of the World Health Organization
DRC	Democratic Republic of the Congo
EOC	Emergency Operations Centre
ES	Environmental Surveillance
GPEI	Global Polio Eradication Initiative
IDP	Internally Displaced Person
IMB	Independent Monitoring Board (for GPEI)
IPV	Inactivated Poliovirus Vaccine (type 1, 2 and 3 antigens)
IQR	Interquartile Range
ISIS	Islamic State of Iraq and ash-Sham
KP	Khyber Pakhtunkhwa (Pakistan Province)
LQAS	Lot Quality Assurance Sampling (Survey)
mOPV1	Monovalent Sabin Strain Poliovirus type 1
mOPV2	Monovalent Sabin Strain Poliovirus type 2
mOPV3	Monovalent Sabin Strain Poliovirus type 3
NEOC	National Emergency Operations Centre (Pakistan)
nOPV2	Novel Oral Poliovirus type 2
OPV	Oral Poliovirus Vaccine
OPV2	Oral Poliovirus Vaccine type 2
PHEIC	Public Health Emergency of International Concern (International Health Regulations, 2005)
PV	Poliovirus
PV1	Poliovirus type 1
PV2	Poliovirus type 2
PV3	Poliovirus type 3
RI	Routine Immunization (Essential Immunization Services)
SAGE	Strategic Advisory Group of Experts on immunization (advising WHO on immunization policy)
SIAs	Supplementary Immunization Activities
tOPV	Trivalent Oral Poliovirus Vaccine (Sabin Strain types 1, 2 and 3)
UNICEF	United Nations Children’s Fund (formerly; now simply UNICEF)
UC	Union Council (Pakistan)
VDPV	Vaccine-Derived Poliovirus
VP1	Viral Protein 1 (Poliovirus Capsid Surface Protein)
WHO	World Health Organization
WPV	Wild Poliovirus
WPV1	Wild Poliovirus type 1
WPV3	Wild Poliovirus type 3
