# Correction: Wassilak et al. Impediments to Progress Toward Polio Eradication During 2014–2024: Effectively Addressing the Current Challenges. *Vaccines* 2025, *13*, 1060

**DOI:** 10.3390/vaccines13121217

**Published:** 2025-12-01

**Authors:** Steven G. F. Wassilak, Abdinoor Mohamed, John Paul Bigouette

**Affiliations:** 1 Independent Researcher, Atlanta, GA 30307, USA; 2 Global Immunization Division, Centers for Disease Control and Prevention, Atlanta, GA 30329, USA; wyr5@cdc.gov (A.M.); qdz1@cdc.gov (J.P.B.)

The authors would like to make the following corrections to this published paper [1]: In the original publication, there was a mistake in Figure 1 as published. Some areas in Pakistan were labeled incorrectly when the figure was transformed to a single-object picture. The corrected Figure 1 appears below.

In addition, in the third sentence of Section 4.2 regarding vaccine-derived polioviruses, the year ‘2020’ should have been ‘2000’; the correct sentence is as follows: “However, such outbreaks may have been few: before 2000, natural immunizing infections with WPVs may have prevented wide circulation of Sabin-related poliovirus [146].”

The authors state that the scientific conclusions are unaffected. This correction was approved by the Academic Editor. The original publication has also been updated.

## Figures and Tables

**Figure 1:**
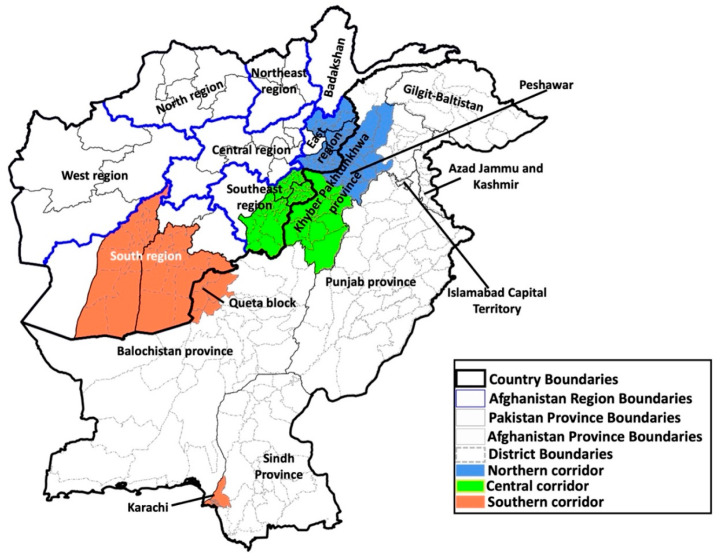
Regions/provinces of Afghanistan and Pakistan, critical districts and cities affected by poliovirus transmission, and intercountry transmission corridors—2014–2024.
